# Harnessing Different Motivational Frames via Mobile Phones to Promote Daily Physical Activity and Reduce Sedentary Behavior in Aging Adults

**DOI:** 10.1371/journal.pone.0062613

**Published:** 2013-04-25

**Authors:** Abby C. King, Eric B. Hekler, Lauren A. Grieco, Sandra J. Winter, Jylana L. Sheats, Matthew P. Buman, Banny Banerjee, Thomas N. Robinson, Jesse Cirimele

**Affiliations:** 1 Department of Health Research and Policy, and Stanford Prevention Research Center, Department of Medicine, Stanford University School of Medicine, Stanford, California, United States of America; 2 School of Nutrition and Health Promotion, Arizona State University, Phoenix, Arizona, United States of America; 3 Stanford Prevention Research Center, Department of Medicine, Stanford University School of Medicine, Stanford, California, United States of America; 4 School of Engineering, Stanford University, Stanford, California, United States of America; 5 Department of Pediatrics, and Stanford Prevention Research Center, Department of Medicine, Stanford University School of Medicine, Stanford, California, United States of America; Universidad Europea de Madrid, Spain

## Abstract

Mobile devices are a promising channel for delivering just-in-time guidance and support for improving key daily health behaviors. Despite an explosion of mobile phone applications aimed at physical activity and other health behaviors, few have been based on theoretically derived constructs and empirical evidence. Eighty adults ages 45 years and older who were insufficiently physically active, engaged in prolonged daily sitting, and were new to smartphone technology, participated in iterative design development and feasibility testing of three daily activity smartphone applications based on motivational frames drawn from behavioral science theory and evidence. An “analytically” framed custom application focused on personalized goal setting, self-monitoring, and active problem solving around barriers to behavior change. A “socially” framed custom application focused on social comparisons, norms, and support. An “affectively” framed custom application focused on operant conditioning principles of reinforcement scheduling and emotional transference to an avatar, whose movements and behaviors reflected the physical activity and sedentary levels of the user. To explore the applications' initial efficacy in changing regular physical activity and leisure-time sitting, behavioral changes were assessed across eight weeks in 68 participants using the CHAMPS physical activity questionnaire and the Australian sedentary behavior questionnaire. User acceptability of and satisfaction with the applications was explored via a post-intervention user survey. The results indicated that the three applications were sufficiently robust to significantly improve regular moderate-to-vigorous intensity physical activity and decrease leisure-time sitting during the 8-week behavioral adoption period. Acceptability of the applications was confirmed in the post-intervention surveys for this sample of midlife and older adults new to smartphone technology. Preliminary data exploring sustained use of the applications across a longer time period yielded promising results. The results support further systematic investigation of the efficacy of the applications for changing these key health-promoting behaviors.

## Introduction

The major killers of adults in the U.S. and many countries worldwide–non-communicable diseases such as cardiovascular disease, cancer, stroke, and type 2 diabetes–have been demonstrably linked to a small number of key health behaviors, including regular physical activity [1], [2] and, increasingly, prolonged daily sitting time [3], [4]. Relatively modest amounts of moderate-intensity physical activity (akin to approximately 20 minutes per day of brisk walking) are associated with improved health, physical function, and psychological well-being [2]. Yet, the majority of the population is not physically active enough to receive such benefits.

Most evidence-based interventions to promote regular physical activity have used in-person instructional formats delivered in clinical or community settings. Among the constraints of such approaches are staff time, intervention fidelity challenges, transportation and venue costs, and reduced intervention personalization when group formats are applied. Such resource-intensive approaches can be especially problematic for sustaining health behavior change over time.

“Tele-health” and other mediated approaches to health behavior change provide an empirically supported, convenient, and potentially lower-cost alternative for reaching large proportions of the public over a longer period of time [5], [6]. Yet, many of these interventions have continued to require a human delivery interface that can constrain health delivery resources. The advent of mobile communication technologies has created a vast potential for both collecting and delivering time- and context-sensitive health information across broad segments of the population. The growth of mobile phone use across socioeconomic and age strata has been staggering, with 322 million wireless subscriber connections as of June 2012, including 34% in a wireless-only household; www.ctia.org, January 9, 2013). Currently, 85% of American adults own a cell phone and 45% own a smartphone. In addition, smartphone usage is higher among Hispanics (49%) and African Americans (47%) compared to whites (42%) (http://pewinternet.org/Commentary/2012/February/Pew-Internet-Mobile.aspx). In addition, in 2012, for the first time, more than half (53%) of adults aged 65 years and older reported using the Internet or e-mail (http://www.pewinternet.org/Reports/2012/Older-adults-and-internet-use.aspx).

Advances in built-in smartphone activity sensors (i.e., accelerometers) provide a timely and potentially cost-efficient means for enhancing ongoing physical activity participation. In addition to continuous activity monitoring, such devices can be programmed to provide automated, behaviorally and contextually tailored information to facilitate health behavior change throughout the day and across a variety of settings.

There has been an explosion of smartphone health promotion applications (apps), including physical activity apps, which have been projected to be in the thousands (e.g., a search in the Apple App Store for “Health and Fitness” resulted in more than 2000 apps available for the iPhone). Yet, relatively few apps have drawn explicitly from relevant behavior change theory or evidence or have undergone systematic evaluation [7]–[9]. Applications of relevant behavioral theory and evidence can inform the selection and timing of intervention components, thereby increasing the potential effectiveness of smartphone-delivered programs.

This first-generation feasibility study aimed to apply a behavioral science-informed user experience design (BSUED) process [10] in developing smartphone applications to increase regular physical activity and decrease sedentary behavior (e.g., prolonged sitting) in adults who to date have received less attention in this field (i.e., midlife and older adults new to smartphone technology). Initial feasibility for health behavior change was assessed using pre-post assessments via standardized measures, and the study aims were achieved.

## Methods

### Ethics Statement

The Stanford University School of Medicine Human Subjects Institutional Review Board approved this study. All participants provided written informed consent.

### Development of the Applications

#### Overview

An interdisciplinary team of behavioral and exercise scientists, health experts, computer scientists, and engineers collaborated in constructing distinct physical activity smartphone applications using behavioral science-informed user experience design (BSUED) [10] and an iterative user testing process. The BSUED process draws from behavioral science theory in delineating motivational “drivers” of behavior change and constructing intervention strategies to reflect those motivational drivers. Identification of relevant intervention strategies were drawn from the behavioral science evidence base in tandem with experiential information obtained from users as part of the iterative design process.

Three behavior change apps to promote regular physical activity and reduce sedentary behavior, based on three distinct motivational frames drawn from behavioral science theory and evidence, were constructed and iteratively tested. One physical activity/sedentary behavior app applied an *“analytic”* motivational frame that was based on social cognitive theory [11] and self-regulatory principles of behavior change [12]. Among the theoretically and empirically based behavior change techniques used in this app were personalized and quantified goal-setting and behavioral feedback, problem-solving around barriers to behavior change, and informational tips or advice for behavior change.

A second physical activity app applied a *“social”* motivational frame drawn largely from social influence theory and perspectives [13]. Among the theoretically and empirically based behavior change techniques utilized in this app were real-time social normative feedback, social support for behavior change, interactions with and modeling of behaviors by similar others (i.e., homophily) [14], and group-based competition and collaboration.

A third physical activity app applied an *“affective”* motivational frame drawn from operant conditioning principles [15], [16] and emotional transference to an avatar, whose movements and behaviors directly reflected the physical activity and sedentary levels of the user. Among the behavior change techniques used in this app were positive reinforcement (i.e., the pairing of a positive reward following a desired behavior), the use of an avatar as a visual model corresponding to self-based performance to provide real-time feedback on progress, and game-like feedback and “jack-pot” rewards contingent upon reaching behavior change milestones.

After arriving at the three behavioral frames, a “design thinking” approach was used to rapidly iterate through concept exploration, prototypes, and ethnographic testing of the user-application interfaces to inform the interaction design architecture for the three apps.

In addition to the above three behavior change apps, an app to compile, analyze, and integrate the built-in accelerometer data being collected on a continuous basis from the project smartphones with the intervention apps was developed. This app, which was programmed to provide “just-in-time” feedback to users of all three behavior change apps using algorithms based on the national recommendations for physical activity (i.e., 150 minutes or more per week of moderate-intensity physical activities such as walking) [2] and scientific evidence related to prolonged sedentary activity [2], was systematically tested and validated against standard external accelerometry (i.e., Actigraph GT3x) under both laboratory- and free-living extended monitoring conditions [17].

### Participants and Procedures

The target population consisted of community-dwelling adults ages 45 years and older who were insufficiently physically active (i.e., engaged in less than 60 minutes of moderate or more vigorous physical activity per week that increased heart rate, breathing, or perspiration), reported typically sitting for 10 or more hours per day, were able to participate safely in a physical activity program based on responses to the physical activity readiness questionnaire [18], and were currently using a mobile phone but not using a smartphone.

#### Initial app development and formative testing

During the period from January 2010–March 2011, the interdisciplinary project team undertook initial app design, programming, and iterative user testing. Twelve individuals meeting the project eligibility criteria described above participated in formative evaluation and user testing of the project apps. Among the activities initiated during this phase of the project was determining the most powerful motivational frames to test based on the behavioral science literature and user interviews, along with the most relevant evidence-based behavior change strategies and techniques accompanying each frame [10]. We then conducted informal semi-structured interviews with the target group to understand possible opportunities and likely barriers to using a smartphone for promoting increased physical activity and reduced uninterrupted time spent sitting [19]. Following these activities, we developed initial app prototypes, obtained feedback from potential users, and iterated on the apps through the use of a variety of low-fidelity prototypes (e.g., sketches and diagrams of concepts developed in a parallel fashion based on previous research on prototyping) [20], [21], mid-fidelity prototypes (e.g., paper prototypes outlining the interactions a person would experience when using the apps), and high-fidelity prototypes (e.g., fully functional systems tried out by user testers over a two-week period). These activities helped to delineate if the concepts and final apps had acceptable usability and theoretical fidelity [22].

The Android smartphone platform was utilized in light of its capabilities with respect to “live wall paper” displays, ease of programming, and ability to run the continuous built-in accelerometer in the background simultaneous with other apps. To prolong the smartphone battery life sufficiently to allow for continuous accelerometer data capture throughout the day, the phone's default battery was replaced with an extended life battery. For all three apps, the data being collected via the smartphone's built-in accelerometer were available within a smartphone-based database for use by the three apps to provide individualized feedback throughout the day. These data were transmitted, via an encrypted protocol, to the project's local servers each evening for data storage and to allow researchers to monitor the quality of data while the study progressed (see below).

The behavioral components for the three apps are summarized in **Table 1**. The three behavior change apps shared the following structural and behavioral elements in common: a glance-able display providing “just-in-time” feedback of the user's current daily physical activity/sedentary activity levels in a visual display format commensurate with the motivational frame of the specific app being used (see below); passive activity assessment throughout the day via the smartphone's built-in accelerometer; brief daily self-monitoring of physical activity and sedentary behavior levels and contexts through the use of previously validated ecological momentary assessment (EMA) questions queried via the smartphone [23]–[25], which users were prompted to complete at the end of each day; and a “help” tab for each app. In addition, for all three apps a major focus was placed on health-enhancing moderate-intensity physical activities undertaken in episodes lasting at least 10 minutes. Brisk walking, in particular, is an appropriate moderate-intensity activity that is preferred by many adults and can be engaged in throughout the day [2]. Hours of sedentary activities per day also were incorporated into the personalized feedback delivered by each app. For adults in this age group, discretionary sedentary activity time is comprised to a large extent of sitting time accompanying television viewing [26].

**Table 1:** Behavioral Components for the Analytic, Affect, and Social Applications.

Components	Smartphone Applications		
	Analytic	Affect	Social
“Push” component (i.e., notifications)	X	X	X
“Pull” component (i.e., information found via participant selecting an icon)	X	X	X
“Glance-able” display	X	X	X
Passive activity assessment	X	X	X
Real-time feedback	X	X	X
Self-monitoring	X	X	X
“Help” tab	X	X	X
Goal-setting	X		
Feedback about goals	X		
Problem-solving	X		X
Reinforcement	X	X	X
Variable interval reinforcement schedule		X	X
Attachment		X	
“Play”		X	
“Jack pot” random reinforcement		X	
Social norm comparison			X
Competition/collaboration			X

**Figure 1:**
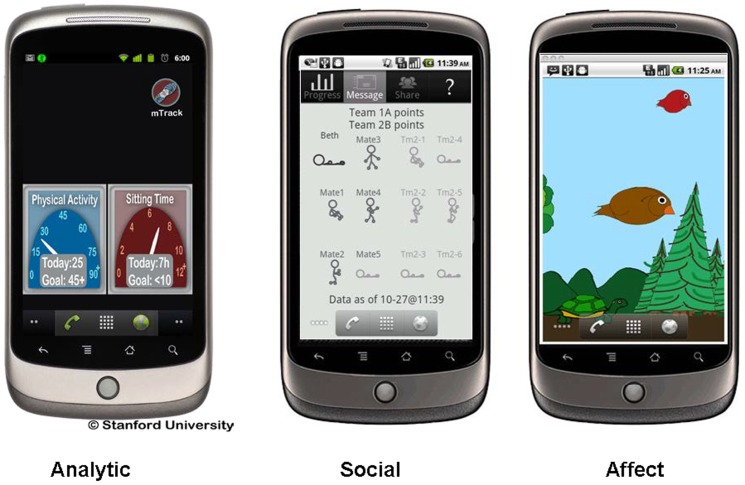
Live wallpaper graphics for the Analytic, Social, and Affect applications.

In addition to the basic elements described above, each of the three behavior change apps had distinct elements based on the theoretical and empirical literature underlying the specific motivational frame being applied. For the *analytic app*, these distinct elements included: a) user-specific goal-setting occurring weekly by the participants which emphasized increasing moderate-to-vigorous physical activity (MVPA), decreasing sedentary behavior, or both based on the individual's preference. For each week a more distal goal to be reached by the end of the study (i.e., an increase to 150 minutes per week in MVPA) [2] or 10% reduction in daily sedentary time [27]) was used as a reference point. Participants were provided with three goal options of varying difficulty (e.g., 30, 60, or 90 minute per week increase in MVPA, or a 10, 20, or 30 minute per day decrease in sedentary behavior) or were allowed to enter their own personal goals. These choice options were given based on a desire to establish graded goals to increase personal self-efficacy while also capitalizing on the utility of the default option aimed at “nudging” individuals towards these graded goals [28]; b) numerical physical activity and sedentary activity feedback related to user goals displayed throughout the day on the smartphone's home screen (see **Figure 1**); c) problem-solving information and advice if users did not meet their goals for a given week (i.e., establishment of a new customized lower goal based on prior goals that were met and additional referencing of the app's informational “tips” for specific advice related to problems the person may be experiencing with meeting their goals); d) text-based positive reinforcement statements that appeared on the phone when individuals either met their weekly goal, exceeded their weekly goal, or met the overall study goal; and e) a history of prior physical activity and sedentary behavior levels displayed graphically.

For the *social app*, additional distinct elements included a live wallpaper display of individual avatars representing the user and other study participants randomized to use this app (using pseudonyms that protected user confidentiality) who had been assigned to the user's “virtual group” as well as the members of a second “virtual group” that did not include the user (see **Figure 1**). The posture displayed for each of the avatars reflected how active each was up to that portion of the day (i.e., lying prone reflected the highest levels of inactivity while a running posture reflected the highest levels of physical activity). Feedback relating to how active/sedentary the user was during the day was displayed in tandem with feedback reflecting the user's group as a whole and feedback about the other virtual group (i.e., social norm comparisons and contextualization). Two virtual “confederate” participants were included for each of the two groups to promote a positive descriptive norm for the group, allow for early participants to have a “group” to join given that participants were enrolled on a continuous basis, and to initially model the posting of content to the message board, described below. (Note that this procedure was approved by the institutional IRB approving the project, and all participants randomized to the social app were informed of it at the completion of the study, according to IRB protocols). Related to promoting descriptive norms [29], while random variability in each confederate was created, over the course of the week each confederate pair for each group averaged 150 minutes per week of moderate to vigorous intensity physical activity and 8 hours of sedentary time per day to ensure equality across the groups. This average for the confederates also implicitly established a descriptive norm metric that was in line with the study goals being utilized within the analytic app.

Similar to the analytic app, a history tab was also available whereby participants using the social app could see a visual summary of the overall history of their physical activity and sitting time. In contrast to the analytic app, however, all personal data in the social app were displayed in reference to group averages (e.g., for daily physical activity level, a participant would see one line representing his/her physical activity for that day along with two other lines in the graph representing the average physical activity for his/her own group and the other group). This history configuration was used to further emphasize the social comparison aspects of this app.

A participant electronic “message board” was also available to users of the social app, where participants could post, in real-time, comments, suggestions, or other information they deemed appropriate to the other individuals assigned to the social app. The message board was not officially moderated (i.e., all posts were immediately shared with others participating in the social app arm of the study). However, to positively influence the injunctive norms and etiquette within the message board, study confederates posted, at least weekly, comments and information that included similar types of material that were incorporated into the informational “tips” and problem-solving strategies used in the analytic app, but written in a style that would conform with the conversational language typically used on electronic message boards. When participants were randomized to the social app, they were given instructions on how to post to the message board and were asked to post a brief introduction of themselves, without divulging any identifying information (e.g., name, exact age, address, phone number, etc.) to ensure confidentiality.

For the *affect app*, an avatar in the form of a bird was used to reflect how active/sedentary the user was throughout the day. The avatar, which appeared on the smartphone's glanceable display throughout the day, changed posture, position, and movement depending upon how active the user was up to that point in the day (see **Figure 1**). Parallel to the goal-related information being received by participants in the analytic and social apps, the visual representation of the bird reflected participant physical activity/sedentary activity behaviors in relation to the overall behavioral goals of the study. Specifically, the bird did not appear to be “happy” until at least 30 minutes per day of moderate-to-vigorous physical activity occurred (a daily level complementary to the 150 minute/week recommendation listed above; [2]) and/or trajectories of sedentary time were commensurate with engaging in 8 hours or less of sedentary time per day. Once the participant surpassed these daily levels, additional levels were accessible and were reflected in further behaviors undertaken by the bird. In particular, in higher stages the bird would become “playful” by first moving toward and following a person's touch on the screen and in advanced stages engaging in additional movements. To foster increased emotional attachment to the bird, there was a “play” screen whereby individuals could, for example, “play fetch” with the bird by “throwing” a virtual stick for the bird to fly over and “catch” and then bring back to the participant.

Positive reinforcement occurred in two ways. First, whenever a person reached pre-specified thresholds related to physical activity or trajectories of sedentary behavior (e.g., through engaging in “breaks” from prolonged sedentary behavior throughout the day), the bird would appear on the live wallpaper and give the person a “thumbs up” while making a melodious sound. These “reinforcers” were delivered on an expanding variable interval reinforcement schedule, demonstrated in the literature to be useful for promoting ongoing maintenance of behavior [15]. In addition, larger “jackpot”-type reinforcers could be earned in the form of extended vocalizations and unexpected arrivals of the bird at different locations that were progressively farther away from the San Francisco Bay Area (e.g., the Eifel Tower in Paris). All of the reinforcers were delivered based on the combination of user physical activity/sedentary activity levels measured via the smartphone's built-in accelerometer and delivered immediately after reaching pre-specified activity thresholds.

Similar to both the analytic and social app, there were options for the participant to view his/her progress and history. Specifically, with each progressive activity level increase, the bird flew higher up on the screen. The highest level attained on the screen was used as a visual “history” of activity across the day. In addition, as the participant accumulated more daily activity, following which the bird reached more distant destinations of interest, pictures of those destinations were added to a “travel” tab whereby all of the places the bird visited were displayed.

#### Feasibility and fidelity testing of the apps

Following the app development activities, we conducted feasibility and fidelity testing of the three apps with respect to their capabilities for impacting initial physical activity and sedentary activity levels. We recruited participants in two waves (n for wave 1 = 27; n for wave 2 = 41). For each wave, individuals meeting the eligibility criteria were randomly assigned, using a computerized version of the Efron procedure [30], to use one of the three custom apps for an 8-week period. Participants were recruited via community advertising including word of mouth, email list-serves, and local forum bulletin boards. The first week of the 8-week period was used as a baseline period during which time only the activity-monitoring app was installed on the study smartphones provided to participants, without a behavior change app. Participants were requested to continue with their normal physical activity and sedentary behaviors during the baseline week. Because the participants in this study had not previously used a smartphone, the initial one-on-one 1-hour training session was used to provide participants with instruction on the general use of the smartphone, including wearing it attached to their waist to optimize accurate data capture via the phone's built-in accelerometer. At the end of this initial week, participants returned to the research facility to receive their randomly assigned behavior change app and basic instruction on its use. In addition to having access to the “help” tab as part of each app, participants could call project staff with any technical problems or difficulties with the apps during the 8-week project period. In addition, written instructions for the smartphone were provided in the form of the manufacturer's user manual along with simplified user instructions designed by the research team to highlight key features of both the smartphone and the apps.

### Measures

To assess the feasibility of increasing regular physical activity and decreasing sedentary activity throughout the day using the three apps, participants completed standard self-administered questionnaires at baseline and at the end of the 8-week intervention period. These questionnaires were used as the primary assessment instruments in this initial app feasibility and fidelity testing procedure because they are straightforward to collect and analyze, and are among the most commonly used outcome measures in the physical activity/sedentary behavior field for the target age group. Using such measures also allowed us to compare the behavior changes observed using our apps to those reported in other physical activity intervention studies, including those using other information technologies, in the same age group.

To assess physical activity levels, the CHAMPS Physical Activity Questionnaire was used. This instrument has been found to provide a valid and reliable estimate of usual physical activity behavior, including walking, in middle- and older-aged adults [31], [32]. Three-month stability coefficients are in the .70–.84 range in community samples of older adults [32]. The instrument has also been shown to have concurrent validity when compared with interviewer-collected physical activity data [32], as well as sensitivity to change in a number of community samples of midlife and older women and men [5], [31], [33]. Given the intervention focus on moderate-intensity activities such as brisk walking and related activities, in light of their particular importance for health and well-being [2], the brisk walking and total moderate-to-vigorous intensity variables were of particular interest.

To assess sedentary behavior levels, the Australian sedentary behavior questionnaire (referred to as the Measure of Older Adults' Sedentary Time [MOST]) was used [34]. This measure has been shown to have acceptable test-retest reliability (i.e., Spearman Rho = 0.52–0.90), and has been shown to be efficacious at detecting change within intervention studies [27], [34]. The measure includes metrics for a variety of sedentary behaviors such as television viewing, reading, or office work and metrics for each individual behavior along with total sedentary time have been developed. Given that television viewing is the most prevalent discretionary sedentary activity undertaken by people in this age group [26], television-viewing time was considered to be the primary sedentary variable of interest.

To evaluate user acceptability of the apps, participants completed a user satisfaction survey at the end of the 8-week intervention period. The survey, adapted from similar user satisfaction surveys in this age group [35], consisted of 22 items asking users to rate, on a 6-point Likert-type scale, level of disagreement to agreement with each item concerning the usability of the apps. An additional 20 items captured participants' general attitudes towards smartphones following the intervention period on a 5-point Likert-type scale (adapted from Nickell and Pinto, 1986) [36].

### Data Analysis

To evaluate the feasibility of each app for improving initial physical activity and sedentary behavior patterns, pre-post paired-comparison *t* tests were conducted for each app on mean moderate-intensity (brisk) walking levels and moderate-to-vigorous (MVPA) physical activity variables from the CHAMPS questionnaire and the mean hours of daily television viewing time from the Sedentary Behavior Questionnaire. Because initial analysis indicated no significant differences by recruitment wave, data were combined in presenting the results. Analysis of covariance was used to explore between-group differences in the variables of interest across apps, with all major outcome variables log-transformed in response to non-normality. Descriptive statistics were obtained on the post-intervention user satisfaction survey and, for presentation purposes, summarized as percentages of participants who agreed vs. disagreed with each of the items assessed.

## Results

### Description of Participants

The 68 adults participating in the intervention app feasibility testing protocol were an average of 59.1±9.2 years old (range = 45–81 years), with 73.5% women. Seventy-six percent had a college degree, 51.4% had an annual household income of $70,000 or greater, 48.5% were working full-time, and 39.7% reported being currently married. Sixty-nine percent were non-Hispanic White, 13% were Hispanic/Latino, and 12% were Asian. Mean body mass index (BMI) was 29.6±6.2. A third of the sample was randomized to each smartphone intervention app (Analytic n = 22; Social n = 23; Affect n = 23), with no significant between-group baseline differences found in the demographic variables or baseline physical activity or sedentary behavior variables (*p* values >0.05). The sample's mean baseline brisk walking minutes per week from the CHAMPS questionnaire was 79.9±92.3 (range = 73.7–88.6 across apps). The sample's mean daily television viewing time was 2.3±1.5 hours per day (range = 1.9–2.5 across apps).

While all but one participant was successful in using their assigned smartphone app through at least 5 weeks of the 8-week protocol, 7 participants were missing post-test physical activity or sedentary behavior questionnaire data (i.e., 10.3%). Missing questionnaire data were due to participant time constraints or not properly filling out the questionnaires. Within the constraints imposed by analysis of subgroups with small n's, independent *t*-tests or Chi-Square analyses comparing the 7 participants with missing post-test questionnaires with the rest of the sample indicated that the 7 participants were significantly different than the full sample with regard to age (dropouts = 52.3±10.5, completers = 60.0±8.8, *p*<0.05) but not significantly different from the rest of the sample in other demographic variables (i.e., gender, race, education, income), BMI, group assignment (N missing: Analytic = 3; Social = 2; Affect = 2), or baseline physical activity or sedentary behavior variables (*ps*>0.08).

### Changes in Moderate-to-Vigorous intensity Physical Activity

Participants across all three apps reported significant mean increases in weekly minutes of brisk walking across the 8-week intervention period (paired *t*_[60]_ = 5.3, *p*<0.0001) (between-group difference non-significant, *p*>0.73). The increase in weekly minutes of brisk walking across the three apps averaged 100.8±167.0 minutes (Group Mean minutes/week increase±SD: Analytic = 71.1±147.3; Social = 122.9±153.3; Affect = 105.7±187.2). Similarly, participants across all apps reported significant mean weekly increases in total moderate-to-vigorous physical activities (paired *t*_[60]_ = 4.5, *p*<0.0001) (between-group difference non-significant, *p*>0.99). The increase in weekly minutes of moderate-to-vigorous physical activity across the three apps averaged 188.6±289.3 minutes/week (Analytic = 172.9±200.5; Social = 257.1±323.8; Affect = 134.3±319.1).

### Changes in Discretionary Sitting Time

Study participants also reported significant decreases in the daily amount of discretionary time they spent sitting in front of the television (paired *t*_[58]_ = 2.5, *p*<0.02) (between-group difference non-significant, *p*>0.34). The decrease in daily minutes of television viewing time averaged 29.1±84.5 minutes/day across the three apps. While the between-group difference in this variable was non-significant (*p*>0.34), the mean decreases appeared to be larger in the Analytic and Social apps relative to the Affect app (mean for Analytic = 48.9±81.7; Social = 34.9±95.1; Affect = 6.5±74.3).

### Post-Intervention User Satisfaction with the Apps

In general, participants reported positive experiences with the three apps. The majority of the sample (87%) reported that they found the apps easy to use; 77% reported that the length of time needed to use the apps “was about right”, and only 11% reported that the number of contacts with the apps “was too many”. Only 16% reported having a hard time remembering to use the apps. Over two-thirds of participants (69%) reported that the apps motivated them to be more physically active and to sit less (74%), and the majority of participants reported that the apps helped them remember to exercise regularly (71%) and made them aware of their sitting time (87%).

After using the smartphone apps for an 8-week period, this initially smartphone-naïve sample of midlife and older adults reported generally more positive than negative attitudes related to smartphones in general. For example, 91% agreed that smartphones are a fast and efficient means of gaining information, and 85% agreed that smartphone applications have unlimited possibilities that have yet to be thought of. Relatively few participants reported that smartphones made them uncomfortable because they did not understand them (9%), or were intimidating because of their complexity (18%). No significant between-app differences were discerned, but small sample sizes reduced power to detect such differences.

With respect to difficulties with the apps reported during the study, we found such user difficulties to be, for the most part, relatively minor and readily resolved. The most common difficulties experienced by users included questions concerning whether the app was registering physical activity consistently (44%; after ensuring that the app was working properly, staff checked the phone to ensure that it was being used and worn properly, and participants were provided with some additional instructions in phone use and the importance of attaining the moderate-intensity or more vigorous levels of activity upon which the feedback was based); reports that the phone with the extended battery was heavy to carry (29%; participants were given carrying pouches or belts on which to attach the phones if they did not have appropriate ones); and reported difficulties using some of the general smartphone features (e.g., making and receiving calls, retrieving voice mail, etc.) (23%; staff produced simplified instruction sheets addressing the most frequently asked questions in this area). Typical of mobile phone use more generally, 18% reported dropped calls or poor mobile phone coverage from time to time, and 9.5% reported some difficulty reading the mobile phone screen. None of the above difficulties led to participant loss to follow-up.

### Continued use of Apps following the Study Period

To begin to explore how long participants would be willing to continue using the custom apps following the 8-week study period, 12 participants enrolled in the latter stage of the study (4 from each app group) were allowed to continue to access their assigned app if they so chose until the investigators collected all smartphones on day 233 post-study. Participants were approached in consecutive order just prior to their 8-week study end date and were invited to continue using their assigned apps until the number that agreed reached 12 (4 from each app group). Of the 15 participants approached, 80% were willing to continue using their assigned app. The reason the three participants gave for declining further participation concerned their disinclination to continue being “connected” this intensively to their mobile phones.

These 12 participants continued to use their apps for a mean of 191±33 days post-study (range = 120–233) (Analytic: M = 211.0±19.0 days; Social: M = 199.3±27.8 days; Affect: M = 162.0±33.5 days). Over half (53.5%) of these participants, who completed an additional user satisfaction survey at the end of this maintenance period, indicated that they would be willing to use their assigned app for an additional 6 months or longer. In addition, 70% indicated that they would recommend the app to others.

## Discussion

While there has been a steady rise in mobile device applications aimed at promoting regular physical activity and related health behaviors, few have drawn systematically from behavioral science theory or evidence. This first-generation feasibility study applied a behavioral science-informed user experience design (BSUED) process [10] to develop three distinct smartphone applications (apps) using three different motivational frames to increase moderate-to-vigorous physical activity and decrease sedentary behavior. Eight-week feasibility testing of the 3 apps in smartphone-naïve, initially sedentary midlife and older adults indicated that the apps were sufficiently potent to significantly increase average minutes per week of brisk walking and general levels of moderate-to-vigorous physical activity. The mean sample increase in weekly minutes of brisk walking and moderate-to-vigorous physical activity compares favorably with increases observed in controlled trials of communication technology-based physical activity interventions in similarly aged initially inactive adults from the same region and using the same measurement instrument [24], [35], [37].

The apps also appeared to be useful for decreasing the average number of daily minutes participants spent sitting in front of the television—a highly prevalent discretionary sedentary behavior among individuals in this midlife and older age group [26]. The average decreases observed among the three apps, however, suggested that the Affect app was less successful in changing this type of sedentary behavior relative to the other two apps. A larger study is indicated to better evaluate this possibility. In contrast to the other two apps, the Affect app did not provide specific numerical or graphical information that reminded individuals about their physical activity and sedentary behavior targets. Instead, the feedback received in viewing the bird avatar in the Affect app was more representative of the individual's general amount of movement throughout the day (as opposed to more specific information about physical activity and sedentary time). Thus, it is possible that more explicit information may be necessary to obtain significant decreases in this type of sedentary behavior in this age group.

We found all three apps to be generally easy to use and acceptable by the current sample of participants, who had no prior experience with smartphones. Given participants' initial levels of inactivity, careful instruction on the overall physical activity goals targeted in the three apps, i.e., accumulating physical activity of at least moderate intensity (akin to brisk walking) for 10 minutes or more at a time, occurred at the beginning of the interventions and when participant questions arose concerning the feedback they were receiving from the apps.

While the sample was well educated, 25% were from racial/ethnic minority groups, a significant proportion were women, and all were from the aging population segment—groups that traditionally have been under-represented in information technology-based health behavior research [38].

Among the methodological limitations of this study is the lack of an appropriate control group against which to directly compare the effects of the three smartphone apps. The next step in this line of research is to investigate systematically the efficacy of the smartphone apps relative to such a control [39]. Another study limitation is the relatively small sample size. Other aspects that deserve systematic investigation include assessing changes with objective measures of physical activity and sedentary behavior, increasing the diversity and age range of the sample under study (e.g., adolescents may be a useful age group to target using these types of smartphone apps), and extending the time period of app use. While 8 weeks is a reasonable time period in which to investigate initial uptake or adoption of a health behavior [24], [40], longer-term maintenance of health behavior changes is deemed particularly worthwhile to study [41]. This is because there is a dearth of longer-term studies, particularly in the eHealth field [38]. While our initial exploration of this maintenance issue in a small subset of participants was encouraging, further systematic evaluation of this important issue in larger samples is clearly warranted. Evaluation of longer-term use is particularly indicated given that it is possible that the novelty effects generated by such apps could diminish over time, reducing participants' interest in continued use of the apps. While it would also be useful to explore how long the behavior changes might persist following the use of the three apps, a major behavioral objective of developing such apps is to embed them seamlessly into daily smartphone use to allow them to be used indefinitely.

In conclusion, integrating behavioral science theory and evidence with an iterative user-oriented design process may enhance the potency of mobile device applications aimed at promoting behavior change in key health areas such as physical activity and sedentary behaviors. The current results set the stage for systematic investigations of such applications within the context of experimental studies as well as in comparison to commercially available programs.
